# Effect of Modified Manufacturing Conditions on the Composition of Greek Strained Yogurt and the Quantity and Composition of Generated Acid Whey

**DOI:** 10.3390/foods11243953

**Published:** 2022-12-07

**Authors:** Stavros Karastamatis, Evangelia Zoidou, Golfo Moatsou, Ekaterini Moschopoulou

**Affiliations:** Laboratory of Dairy Research, Department of Food Science and Human Nutrition, Agricultural University of Athens, Iera Odos 75, 118 55 Athens, Greece

**Keywords:** Greek yogurt, acid whey, yogurt centrifugation, milk heat treatment

## Abstract

Greek strained yogurt is produced in high quantities worldwide. This production leaves behind acid whey, a by-product that is an environmental challenge. Hence, efforts are made to minimize the acid whey generation. In this study, the combined effect of the different heat treatment levels of milk and the different time of straining on the composition of the produced strained yogurt, as well as on the quantity and composition of the expelled acid whey, was investigated. The initial yogurts were prepared with bovine milk heated at 85 °C/16 s or 100 °C/16 s or 90 °C/5 min, and the acid whey was removed by centrifugation (5500 rpm, 5 min, 25 °C) either immediately after incubation or after 24 h. The results showed that, regardless of the heat treatment of milk, straining after 24 h resulted in an 8% increase in the yield in strained yogurt and about an 11% decrease in the generated acid whey, compared to straining immediately after incubation. The heat treatment level of milk significantly influenced the fat, lactose, and total solids contents of the strained yogurts, as well as the residual whey proteins, protein, and total solids contents of acid whey. Yogurt’s sensory properties were not affected significantly. It was concluded that the quantity of the acid whey expelled during the production of Greek strained yogurt could be decreased without affecting the general quality of the yogurt.

## 1. Introduction

Authentic Greek strained yogurt has been produced in Greece for many decades. According to Greek legislation, this product is obtained from yogurt after straining part of the serum (acid whey) and must contain at least 5.6% protein when it is made from bovine or caprine milk and 8% when it is from ovine milk. In the case of mixtures of different types of milk, the minimum protein content is based on the proportion of the types of milk [1]. Therefore, the term ‘straining’ included in the definition of the Greek strained yogurt, is, in a way, the identity of this product. Straining of the initial yogurt can be achieved by centrifugation or by, filtration through membranes or by filtration through a cloth bag (traditional method) [1,2,3].

Greek strained yogurt is appreciated worldwide as a highly nutritious dairy product because of its high percentage of total solids and especially in protein content. For this reason, not only the production of strained yogurt in Greece but also the production of similar products that are produced abroad, known as Greek Yogurt or Greek-style Yogurt, are constantly increasing. However, the technology procedure for such products is not the same. Except for the straining of the yogurt coagulum, as mentioned before, the high total solids content may be achieved by fortification of the yogurt milk with milk solids, i.e., product formulation. Tamime et al. [2] have extensively reviewed the strained fermented milk products that exist worldwide and have shown substantial differences in their composition, i.e., sugar, fat, and protein contents may range between 1 and 12, between 0 and 20, and between 3.3 and 11%, respectively.

Acid whey, the by-product of Greek strained yogurt manufacture, has pH 3.5–4.5 and may contain 5–7% total solids, 0.03–0.38% fat, 3.37–4.99% lactose, 0.17–0.66% protein, 0.5–0.7% ash, and 0.6–1.40% lactic acid [4,5,6,7]. It has been reported that in order to produce 1 kg of Greek strained yogurt, 2–3 kg of acid whey is left behind, and obviously, this fact leads to environmental challenges [8]. The elimination degree of acid whey, which is actually the degree of separation of the liquid phase from the yogurt gel, i.e., the syneresis phenomenon, depends on the water holding capacity (WHC) of the initial yogurt and is strongly affected by the ratios of protein to total solids and of whey protein to casein. Thus, in order to minimize syneresis or, in other words, to increase the WHC of the yogurt gel, the total solids and protein content should be increased to retain water, particularly in the protein matrix. Moreover, the use of stabilizers helps to reduce syneresis and hence to minimize acid whey generation [3,9]. Regarding the effect of heat treatment of milk in yogurt production, it is known that one of the purposes is the denaturation of whey proteins, which affects the acid whey generation since denatured whey proteins increase the WHC of the yogurt. For yogurt production, milk is usually heat treated at 90 °C to 95 °C for 5 to 10 min [9,10], but the ESL process, i.e., heating at 125 °C to 140 °C for 1–10 s has also been studied. Thus, Savello and Dargan [11] showed that syneresis was significantly reduced in yogurt made from ultrafiltrated skimmed milk and heated at 100–130 °C, while Ichimura [12] reported that the combination of heat treatment of milk and 35 + 5 MPa homogenization improved the texture properties of the yogurt. Finally, Domagala [13] found that syneresis of yogurts prepared from caprine, bovine, or ovine milk decreased substantially after 14 days of storage.

In literature, there are few studies that have been made with the scope to minimize the generation of acid whey. The use of hydrocolloids to limit the acid whey volumes during the Greek yogurt production was reviewed by Gyawali and Ibraim [14], who suggested the use of pectin 0.05% (*w*/*v*) and whey protein concentration (1% *w*/*v*) to increase the WHC from 33% (in control yogurt) to 56% [14,15]. The concentration of milk by ultrafiltration may result in about 78% less acid whey removal than the non-concentrated milk [16], while the increase in the total solids in the yogurt milk initially to 20% by adding milk protein concentrate and straining traditionally through a cloth bag also reduces the whey generation [4]. These studies were based on the product formulation or on the use of ultrafiltrated milk or stabilizers.

The aim of this study was to investigate the combined effect of the different heat treatment levels of the milk with the different times of straining on the composition of the produced strained yogurt as well as on the quantity and composition of the expelled acid whey.

## 2. Materials and Methods

### 2.1. Yogurt Manufacture

**Figure 1:**
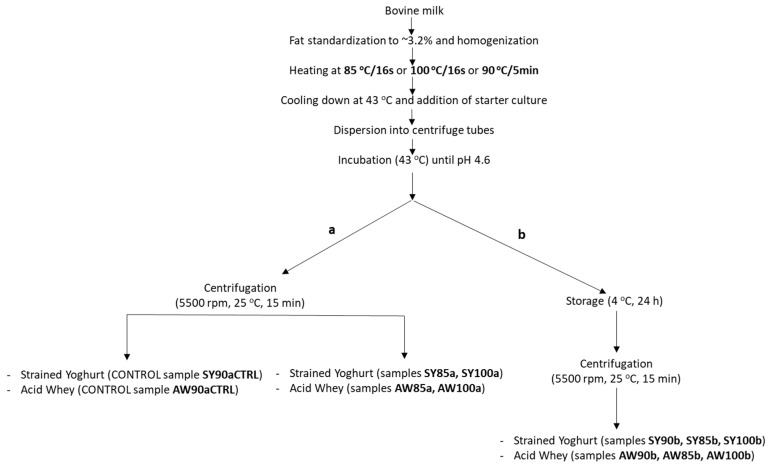
Production of strained yogurt from bovine milk heated at different conditions. Straining took place immediately (**a**) or after 24 h (**b**) after incubation.

Strained yogurts were prepared according to the diagram shown in Figure 1. Bovine milk was standardized at 3.2% fat, homogenized at 250/50 bar at 55 °C, and heated at 85 °C/16 s or at 100 °C/16 s by the continuous indirect method on a laboratory heating system HT220 HTST/UHT (OMVE Lab and Pilot Equipment, 3454 MZ, De Meern, The Netherlands) equipped with a two-stage in-line homogenizer (Twin Panda, Gea Niro Soavi, Type NS2002H, Parma, Italy). Moreover, the same homogenized milk was batch heated in an open vat at 90 °C for 5 min. After heating, each milk was cooled down at 43 °C and was inoculated with a lyophilized yogurt starter culture (Danisko, Yo-Mix 401) diluted in the milk of the corresponding heat treatment at a ratio of 1% (*v*/*v*). Immediately, 40 g inoculated milk was distributed in sterilized 50 mL centrifuge tubes served as yogurt containers and were incubated at 43 °C until a pH value of yogurt 4.6 was reached. After incubation, which lasted about 4 h, half of the yogurts were centrifuged at 5500 rpm at 25 °C for 15 min to remove the acid whey (strained yogurts SY85a, SY100a, and SY90aCTRL), and the other half were stored at 4 °C and were centrifuged under the same conditions after 24 h (strained yogurts SY85b, SY100b and SY90b). Prior to centrifugation, all centrifuge tubes were mechanically stirred to break the gel. The acid whey was collected and filtered to remove any large particles and was kept at −22 °C until analyses, while the strained yogurt was stored at 4 °C. The experiment was carried out in triplicate. Yogurt SY90aCTRL and its acid whey AW90aCTRL were defined as control samples because heating and straining parameters were the typical ones that are applied in the yogurt industry.

### 2.2. Physicochemical Analyses of Strained Yogurt and Acid Whey

The pH of yogurts and of acid whey were measured on a pH meter (WTW multi3420, Germany). The titratable acidity, expressed as g of lactic acid per 100 g of product, was determined in 10 mL acid whey and in 10 g yogurt diluted with 10 g of distilled water, using N/9 NaOH and phenolphthalein as an indicator. Both analyses were performed at the 1-day post-manufacture, i.e., the next day of strained yogurt production. The total solids, fat, and protein contents of yogurts were determined on a FoodScan-Dairy Near Infrared (NIR) analyzer (Foss, Hilleroed, Denmark). Ash content was determined by the AOAC method [17]. The total solids, fat, and protein contents of acid whey were determined on a Milkoscan FT120 (Foss, Hilleroed, Denmark).

Residual sugars and lactic acid contents of the strained yogurt and acid whey samples were determined by HPLC using a Perkin Elmer Flexar system (Shelton, Connecticut USA) and the Aminex HPX-87H, 300 × 7.8 mm ion exchange column (Biorad Inc., Hercules, CA, USA). Sulfuric acid solution 5 mM was used for isocratic elution of 20 μL of sample at 35 °C with a flow rate of 0.5 mL/min [18,19]. The yogurt samples were prepared as follows. In total, 5 grams of yogurt were mixed with 20 mL of sample preparation solution, i.e., solution containing 0.7% sodium tungstate dehydrate, 0.01% orthophosphate, and 7% sulfuric acid 1N, in a 50 mL volumetric flask, the volume was made with ultra-pure water, and then the mix was filtered with filter paper Whatman No. 40. One mL of filtrate was mixed with 0.1 mL 70% perchloric acid, and after storage at 4 °C for 24 h the mixture was centrifuged at 10,000× *g* for 30 min at 4 °C. The supernatant was filtered with a 0.22 μm syringe filter before HPLC analysis. Quantification was based on the respective standard curves. The acid whey samples were prepared as follows: In total, 3 mL of acid whey were mixed with 7 mL of ultra-pure water and were centrifuged as above. Then, to 1 mL of the supernatant, 0.1 mL of perchloric acid 70% was added, and the preparation was continued as described above for the yogurts.

Moreover, acid whey was analyzed for the main inorganic elements. Calcium, magnesium, potassium, and sodium contents were determined on a Shimadzu AA-6800 Atomic Absorption Spectrophotometer (Shimadzu, Kyoto, Japan), according to the ISO/IDF standard method [20]. Phosphorus content was determined by molecular absorption spectrometry [21].

Finally, the effect of the heat treatment on the denaturation of the major whey proteins α-lactalbumin (α-La) and β-lactoglobulin (β-Lg) of acid whey was assessed by the RP-HPLC method described by Moatsou et al. [22]. In brief, a Vydac C4 214 TP 5415 column (Columbia, MD, USA) was used, gradient elution was performed using water-acetonitrile in the presence of trifluoroacetic acid solvents at a flow rate of 1 mL/min, and the eluent was monitored at 214 nm. The quantification of the residual whey proteins was based on the residual chromatographic area of the respective peaks of the milk heated at 85 °C/16 s.

### 2.3. Sensory Evaluation of Strained Yogurt

All strained yogurts were assessed for their flavor, appearance, acidity, white color, odor, viscosity, and presence of aggregates (curds) at the 1-day post-manufacture. The evaluation was performed by a panel of 6 experienced laboratory staff members using descriptive analysis on a 0–10 intensity scale. Samples were presented to assessors in random order.

### 2.4. Statistical Analysis

The effect of the heat treatment and the straining time on the characteristics of strained yogurt and acid whey were assessed by analysis of variance (ANOVA), and the investigation of statistically significant differences was conducted with the Least Significant Difference (LSD, *p* < 0.05). The software Statgraphics Centurion XVI (Manugistics, Inc., Rockville, MA 20852, USA) was used.

## 3. Results and Discussion

### 3.1. Quantity and Composition of Acid Whey

To produce Greek strained yogurt, the necessary processing step is to remove part of the acid whey by various methods. In this experiment, acid whey was removed by centrifugation, and the expelled quantity ranged from 1.8 kg to 2.75 kg/kg of strained yogurt (Table 1). The results agree with those reported by Erickson [8]. Moreover, it is obvious that the quantity of the generated acid whey gradually decreased from the less intense heat treatment to the more intense one. Specifically, initial yogurt made with milk heated at 85 °C/16 s yielded significantly (*p* < 0.05) more acid whey than yogurt made with milk heated at 100 °C/16 s, and the latter significantly more than the control yogurt made with milk heated at 90 °C/5 min at both straining times.

Heat treatment of milk causes significant physicochemical changes, mainly in the fraction of whey proteins, which lead to the formation of a more stable gel with an increased ability to retain moisture [10]. Therefore, the reduced acid whey excretion from the yogurts made with milk treated by batch heating at 90 °C/5 min was due to the more stable gel formed because of, the higher denaturation degree of whey proteins and mainly of the β-Lg as shown in Table 2.

As far as the straining time is concerned, the quantity of the generated acid whey was lower when the centrifugation of initial yogurt took place after 24 h (samples AW85b, AW100b, and AW90b), compared to centrifugation on the same day of production (samples AW85a, AW100a, and AW90aCTRL) regardless the heat treatment of the milk. This difference was attributed to the decrease in the pH of yogurt from 4.6 at the end of incubation to pH 4.2–4.4 at the 1-day post-manufacture. In general, it has been reported that there is a strong correlation between the pH at the end of incubation and the yield of strained yogurt that results after centrifugation. At pH 4.1–4.3, the yield of strained yogurt is higher [23,24]. Finally, the production of 2–3 kg of acid whey for 1 kg of strained yogurt, according to Erickson [8], means that the ratio of acid whey/initial yogurt is about 75 g/100 g. In the present study, 69.5 g and 67 g of acid whey were removed on average from 100 g of initial yogurt immediately after incubation or after 24 h to produce 30.5 g and 33 g of strained yogurt, respectively. Therefore, regardless of the heat treatment of milk, straining at the 1-day post-manufacture of the initial yogurt leads to an 8% increase in the yield in strained yogurt and about an 11% decrease in the generated acid whey, compared to straining immediately after incubation.

Regarding the physicochemical composition, the pH values of acid whey ranged from 4.37 to 4.6 and were in accordance with those reported by other researchers [6,7,25]. Moreover, it is obvious that pH values were not significantly affected by the level of milk heating or by the straining time (Table 1). On the other hand, acidity was significantly affected by the straining time. Concerning the heat treatment of yogurt milk, significant differences were observed between the AW100a and AW90aCTRL in the total solids contents. In addition, the fat content of the acid whey was significantly affected by the time of straining, while the protein content was significantly affected by the heat treatment of milk (Table 1). The differences in the protein content were attributed to the effect of the heat treatment on the whey proteins since, depending on its intensity, it causes changes in their structure. α-La is less heat sensitive than β-Lg that is 33% denatured at 80 °C for 4 s [26]. Acid whey AW85a and AW85b contained more protein because at the heat treatment 85 °C/16 s, the whey proteins were not totally denatured, and hence, those non-denatured, being in their soluble form, lost in the acid whey. As shown in Table 2, the total whey proteins were denatured by about 40% as the temperature increased from 85 °C to 100 °C for 16 s (samples AW100a and AW100b). At the heat treatment of 100 °C/16 s, the denaturation of β-Lg in samples AW100a and AW100b was about 62% and 78%, respectively, whereas the corresponding values of α-La were about 8 and 5.5%. Finally, the batch heating of milk at 90 °C/5 min, which is more severe compared to the heat treatments 85 °C/16 s and 100 °C/16 s, caused denaturation of β-Lg more than 97% in both straining times, and about 76% and 69% denaturation of α-La in the straining time α and β respectively.

As expected, lactose content was lower in the acid whey expelled after 24 h because of its further fermentation by the yogurt culture (Table 3). At the same time, galactose content was higher because of its accumulation since it is not metabolized by the yogurt cultures *S. thermophilus* and *L. delbrueckii* ssp. *bulgaricus* [27]. Galactose content was not affected significantly either by the different heat treatments or by the straining time, and the same was observed for lactic acid. The latter, which is the most important compound of acid whey for its further exploitation, was found in percentages from 0.75% to 0.86% that are similar to those reported by Chandrapala et al. [7]. 

Calcium content was lower in the acid whey obtained from the yogurts strained after 24 h (samples AW85b, AW100b, and AW90b) compared to acid whey obtained from the straining immediately after incubation (samples AW85q, AW100a, and AW90aCTRL) regardless the heat treatment of milk. The same was true for phosphorous content (Table 4). Heat treatment affected the potassium content, and the samples AW90aCTRL and AW90b had the highest concentrations. Calcium content agreed with those reported by Menchik et al. [25], while the same researchers reported phosphorus content between 67 and 69 mg/100 g. However, Kirdar et al. [28] have reported a phosphorus content in acid whey as low as 15.7 ± 2.3 mg/100 mL. The same researchers have found the values 93.4 ± 13.0, 125.7 ± 15.9, 96.9 ± 25.7, and 94.0 ± 10.4 mg/100 mL of acid for calcium, potassium, magnesium, and sodium content, respectively. The corresponding values found by Smith et al. [29] are 123 mg, 147 mg, 11 mg, and 44 mg/100 g of acid whey.

In general, the composition of acid whey was in accordance with those reported by other researchers [5,7,30]. However, Menchik et al. [6,25] have reported 0.17–0.37% total protein content and up to 3.5% lactose content in acid whey from Greek yogurt, whereas Smith et al. [29] have found only 0.02% (*w*/*w*) true protein and 5.6% total solids. The reported differences in the composition of acid whey reflect obviously the variety of the production methods of Greek yogurt.

### 3.2. Composition of Strained Yogurt

The composition of the strained yogurts is shown in Table 5. Strained yogurts SY100a, SY100b, and SY90b had a similar composition to that of control yogurt (sample SY90aCTRL), and those of commercial strained yogurts in Greece that contain 8–10% fat [1] and with those of the full-fat Greek style natural yogurts produced abroad [2].

The acidity of strained yogurts ranged from 1.09% to 1.17%, and these values were higher than the reported values for plain-set-type bovine yogurt [31]. According to Tamime and Robinson [32], the minimum acidity of plain yogurt can be 0.7%, whereas the maximum values of marketed bovine yogurts range from 1.17 to 1.48%. The lower acidity of the strained yogurts SY85b, SY100b, and SY90b compared to their counterparts SY85a, SY100a, and SY90a CTRL (control yogurt) can be attributed to the higher loss of lactic acid to the acid whey (Table 3), although the time of straining did not affect significantly neither the pH and acidity nor the gross composition of yogurts. On the other hand, the heat treatment level significantly influenced (*p* < 0.05) the fat and total solids contents which decreased as the heating temperature increased.

Fat, protein and total solids contents were lower in the yogurts SY85b, SY100b, and SY90b, whereas sugars and ash contents were higher compared to the yogurts SY85a, SY100a, and the control yogurt SY90aCTRL. The composition of strained yogurt is affected by the elimination degree of acid whey. The higher the acid whey elimination, the lower the moisture content and, therefore, the higher the total solids content of the strained yogurt and vice versa. Thus, regardless of the time of straining, the higher total solids, protein, and fat contents of yogurts SY85a and SY85b compared to yogurts SY100a and SY100b, and the latter compared to yogurts SY90aCTRL and SY90b can be attributed to the increased elimination of acid whey observed in this order within these samples (Table 1). Elimination of acid whey is favored by the low pH, and hence the recovery of milk solids (%) in strained yogurt is higher at pH 4.1–4.3, compared to pH 4.5–4.7 [24].

Sugars are soluble in the yogurt serum, and therefore, their higher concentrations in yogurts SY85b, SY100b, and SY90b than in yogurts SY85a, SY100a, and SY90aCTRL (control yogurt) were correlated to the lower amount of the corresponding acid whey which was removed.

### 3.3. Organoleptic Characteristics of Strained Yogurt

**Figure 2:**
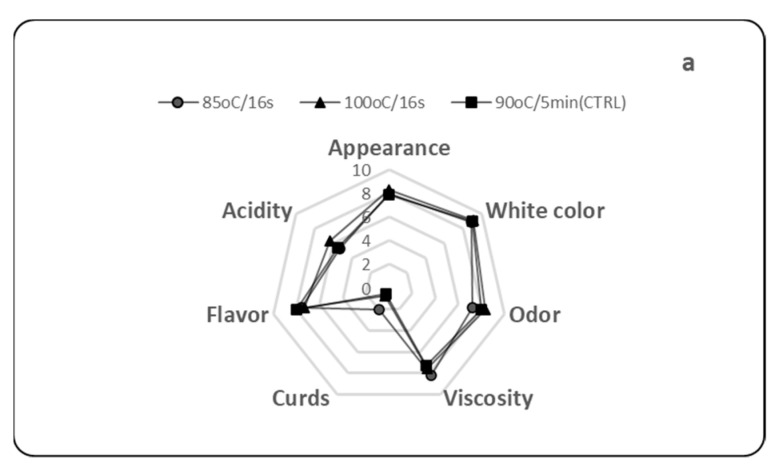

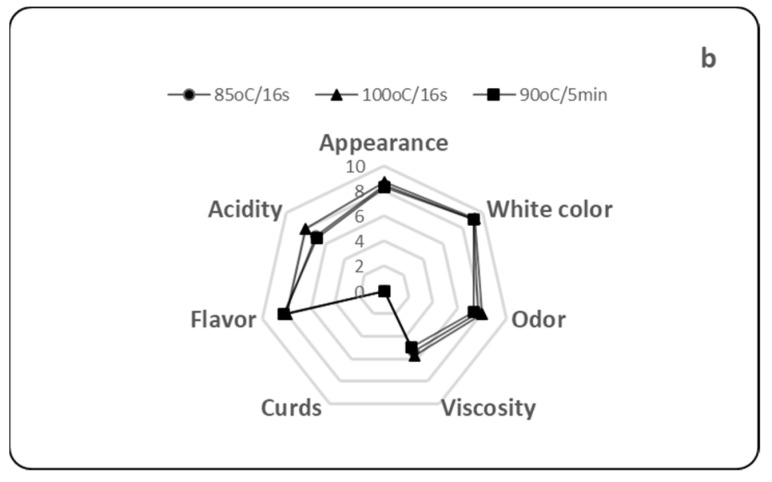
Sensory profiles of Greek strained yogurt from bovine milk heated at 85 °C/16 s or 100 °C/16 s or 90 °C/5 min and strained immediately after incubation (**a**) or after 24 h (**b**).

All strained yogurts were similarly scored for their appearance, color, odor, and flavor regardless of the time of straining (Figure 2). However, the time of straining seemed to affect the acidity, viscosity, and the presence of curds. The yogurts obtained from the straining after 24 h (Figure 2b) were described to have higher acidity and lower viscosity compared to the yogurts obtained when straining took place on the same day of initial yogurt production (Figure 2a).

The lower viscosity of the yogurts obtained from the straining after 24 h was related to the lower total solids content of these yogurts (Table 5), while the higher acidity was related to the lower total solids and fat contents. The titratable acidity is a measure of the buffering capacity and is due to the contribution of milk proteins and anions such as phosphate and citrate and to the lactic acid formed by the fermentation of lactose. However, in yogurt, there is not always a strong relationship between pH and acidity, given the fact that in concentrated yogurt products, pH values as low as 3.7–3.8 do not seem over-acidic because of the high total solids and fat content [33]. The presence of few aggregates (curds) was only in the yogurt, which was made with the milk heated at 86 °C/15 s and strained immediately after incubation. Casein aggregates are formed during incubation, and this defect might disappear if the strained yogurt gel after centrifugation was stirred, a common practice in the industry to incorporate the cream. Finally, no yogurt had an undesirable odor. In a study assessing the organoleptic quality of Greek yogurt, the consumers preferred Greek yogurts with firm, dense texture; moderately sweet aromatic, milkfat, and dairy sour flavors; and moderate sour taste [34].

## 4. Conclusions

From the obtained results concerning the physicochemical composition and the sensory properties of the strained yogurts, as well as the quantity and the composition of the acid whey generated during their production, it is obvious that it may be possible to produce strained yogurt having the typical composition and the consumers acceptance with a parallel decrease in the acid whey volume. This could be achieved if heat treatment of the milk at 90 °C/5 min and a straining of the initial yogurt after 24 h are applied. However, further optimization of the technology parameters may be necessary for scale-up production.

## Figures and Tables

**Table 1:** pH, acidity (%), composition (%), and quantity of acid whey (expressed as kg/kg of strained yogurt) expelled during the manufacture of bovine Greek strained yogurt at different processing conditions (mean ± SD, *n* = 3).

Sample ^†^	pH	Acidity	Fat	Protein	Total Sugars	Ash	Total solids	Quantity
**AW**85**a**	4.55 ± 0.28 ^a,^*	0.45 ± 0.04 ^a^	0.05 ± 0.03 ^a,b^	0.63 ± 0.01 ^a^	4.29 ± 0.16 ^a^	0.69 ± 0.01 ^ab^	5.94 ± 0.10 ^ab^	2.75 ± 0.27 ^a,^*
**AW**100**a**	4.60 ± 0.19 ^a^	0.45 ± 0.02 ^a^	0.04 ± 0.01 ^a,b^	0.51 ± 0.01 ^b,c^	4.31 ± 0.01 ^a^	0.69 ± 0.00 ^a,b^	5.85 ± 0.08 ^a^	2.32 ± 0.04 ^b^
**AW**90**aCTRL**	4.47 ± 0.05 ^a^	0.48 ± 0.01 ^a,b^	0.04 ± 0.01 ^a^	0.41 ± 0.00 ^d^	4.43 ± 0.07 ^a^	0.73 ± 0.01 ^b^	6.10 ± 0.06 ^b^	1.9 ± 0.19 ^d^
**AW**85**b**	4.42 ± 0.08 ^a^	0.50 ± 0.03 ^b^	0.13 ± 0.04 ^c^	0.68 ± 0.05 ^a^	4.29 ± 0.13 ^a^	0.69 ± 0.03 ^a,b^	6.03 ± 0.12 ^a,b^	2.32 ± 0.16 ^b,c^
**AW**100**b**	4.37 ± 0.12 ^a^	0.49 ± 0.02 ^a,b^	0.10 ± 0.03 ^b,c^	0.55 ± 0.03 ^b^	4.22 ± 0.31 ^a^	0.67 ± 0.03 ^a^	5.93 ± 0.14 ^a^	2.04 ± 0.09 ^d,c^
**AW**90**b**	4.37 ± 0.07 ^a^	0.52 ± 0.02 ^b^	0.09 ± 0 ^a,b,c^	0.45 ± 0.02 ^c,d^	4.50 ± 0.08 ^a^	0.70 ± 0.02 ^a,b^	6.10 ± 0.07 ^b^	1.8 ± 0.09 ^d^

^†^ AW85a, AW100a, and AW90aCTRL (control) are the acid whey samples expelled during straining immediately after incubation of yogurts made with milk heated at 85 °C/16 s or 100 °C/16 s or 90 °C/5 min, respectively. ^†^ AW85b, AW100b, and AW90b are the acid whey samples expelled during straining after 24 h. * Means in the same column with different superscripts differ significantly (*p* < 0.05).

**Table 2:** Residual major whey proteins (%) of acid whey expelled during the manufacture of bovine Greek strained yogurt at different processing conditions (mean ± SD, *n* = 3).

Sample ^†^	Total Whey Proteins	α-La	β-Lg
AW85a	100 ^a,^*	100 ^a^	100 ^a^
AW100a	59.53 ± 2.35 ^b^	91.72 ±3.02 ^a^	37.97 ± 1.62 ^b^
AW90aCTRL	11.15 ± 4.46 ^c^	23.84 ± 9.22 ^b^	3.06 ± 0.89 ^d^
AW85b	100 ^a^	100 ^a^	100 ^a^
AW100b	60.46 ± 6.53 ^b^	94.43 ± 10.67 ^a^	21.64 ± 0.36 ^c^
AW90b	13.67 ± 3.53 ^c^	31.16 ± 4.52 ^b^	1.87 ± 0.02 ^d^

^†^ AW85a, AW100a, and AW90aCTRL (control) are the acid whey samples expelled during straining immediately after incubation of yogurts made with milk heated at 85 °C/16 s or 100 °C/16 s or 90 °C/5 min, respectively. ^†^ AW85b, AW100b, and AW90b are the acid whey samples expelled during straining after 24 h. * Means in the same column with different superscripts differ significantly (*p* < 0.05).

**Table 3:** Lactose, galactose, and lactic acid contents (%) of acid whey expelled during the manufacture of bovine Greek strained yogurt at different processing conditions (mean ± SD, *n* = 3).

Sample ^†^	Lactose	Galactose	Lactic Acid
AW85a	3.53 ± 0.21 ^a,b,^*	0.69 ± 0.06 ^a^	0.75 ± 0.08 ^a^
AW100a	3.57 ± 0.04 ^a,b^	0.69 ± 0.04 ^a^	0.76 ± 0.04 ^a^
AW90aCTRL	3.66 ± 0.06 ^a,b^	0.72 ± 0.02 ^a^	0.80 ± 0.02 ^a^
AW85b	3.47 ± 0.06 ^a,b^	0.74 ± 0.06 ^a^	0.81 ± 0.07 ^a^
AW100b	3.41 ± 0.23 ^a^	0.74 ± 0.06 ^a^	0.80 ± 0.04 ^a^
AW90b	3.67 ± 0.04 ^a,b^	0.78 ± 0.05 ^a^	0.86 ± 0.06 ^a^

^†^ AW85a, AW100a, and AW90aCTRL (control) are the acid whey samples expelled during straining immediately after incubation of yogurts made with milk heated at 85 °C/16 s or 100 °C/16 s or 90 °C/5 min, respectively. ^†^ AW85b, AW100b, and AW90b are the acid whey samples expelled during straining after 24 h. * Means in the same column with different superscripts differ significantly (*p* < 0.05).

**Table 4:** Inorganic elements content (mg/100g) of acid whey expelled during the manufacture of bovine Greek strained yogurt at different processing conditions (mean ± SD, *n* = 3).

Sample ^†^	Calcium	Phosphorus	Magnesium	Potassium	Sodium
AW85a	119.32 ± 11.08 ^a,b,^*	56.94 ± 1.86 ^a,b^	15.86 ± 0.58 ^a^	218.43 ± 8.23 ^a^	76.82 ± 13 ^a^
AW100a	116.54 ± 8.70 ^a,b^	53.70 ± 1.71 ^a,b^	15.77 ± 0.58 ^a^	221.49 ± 16.96 ^a^	77.70 ± 0.50 ^a^
AW90aCTRL	124.55 ± 5.86 ^b^	59.85 ± 4.72 ^a^	16.77 ± 0.41 ^a^	258.34 ± 22.25 ^b^	82.63 ± 7.78 ^a^
AW85b	115.80 ± 7.70 ^b,a^	51.84 ± 0.40 ^b^	15.79 ± 0.78 ^a^	229.01 ± 6.21 ^a^	93.86 ± 3.24 ^a^
AW100b	110.25 ± 9.16 ^b,a^	51.89 ± 3.60 ^b^	13,96 ± 1,11 ^a^	222.57 ± 7.29 ^a^	85.05 ± 5.35 ^a^
AW90b	105.71 ± 0.93 ^b^	53.35 ± 2.73 ^b^	15.43 ± 0.83 ^a^	236.34 ± 6.54 ^b,a^	76.46 ± 7.07 ^a^

^†^ AW85a, AW100a, and AW90aCTRL (control) are the acid whey samples expelled during straining immediately after incubation of yogurts made with milk heated at 85 °C/16 s or 100 °C/16 s or 90 °C/5 min, respectively. ^†^ AW85b, AW100b, and AW90b are the acid whey samples expelled during straining after 24 h. * Means in the same column with different superscripts differ significantly (*p* < 0.05).

**Table 5:** pH, acidity (%), and composition (%) of bovine Greek strained yogurt made at different processing conditions (mean ± SD, *n* = 3).

Sample ^†^	pH	Acidity	Fat	Protein	Total Sugars	Ash	Total Solids
**SY85a**	4.37 ± 0.04 ^a,b,^*	1.17 ± 0.02 ^a^	12.44 ± 1.69 ^a^	9.63 ± 1.01 ^a^	2.91 ± 0.03 ^a^	0.65 ± 0.02 ^a,b^	28.31 ± 3.08 ^a^
**SY100a**	4.29 ± 0.07 ^a,b^	1.14 ± 0.02 ^a^	10.92 ± 0.98 ^b,a^	9.16 ± 0.63 ^a^	2.93 ± 0.16 ^a,b^	0.64 ± 0.00 ^a^	25.77 ± 1.94 ^b,a^
**SY90aCTRL**	4.25 ± 0.01 ^a^	1.15 ± 0.10 ^a^	10.13 ± 0.2 ^b^	8.58 ± 0.01 ^a^	3.21 ± 0.07 ^b,c^	0.68 ± 0.04 ^b^	24.66 ± 0.05 ^b^
**SY85b**	4.37 ± 0.06 ^b^	1.13 ± 0.02 ^a^	10.93 ± 0.27 ^a,b^	9.16 ± 0.84 ^a^	3.05 ± 0.09 ^a,b^	0.66 ± 0.01 ^a,b^	25.89 ± 0.77 ^a,b^
**SY100b**	4.43 ± 0.03 ^b^	1.09 ± 0.04 ^a^	10.20 ± 0.42 ^b^	8.74 ± 0.79 ^a^	3.03 ± 0.21 ^a,b^	0.64 ± 0.02 ^a^	24.46 ± 0.77 ^b^
**SY90b**	4.31 ± 0.08 ^b,a^	1.12 ± 0.03 ^a^	9.81 ± 0.52 ^b^	8.73 ± 0.72 ^a^	3.30 ± 0,10 ^c^	0.68 ± 0,03 ^b^	24.34 ± 0.6 ^b^

^†^ SY85a, SY100a, and SY90aCTRL (control) are the strained yogurt samples made from bovine milk heated at 85 °C/16 s or 100 °C/16 s or 90 °C/5 min, respectively and strained immediately after incubation ^†^ AW85b, AW100b, and AW90b are the strained yogurt samples that strained after 24 h. * Means in the same column with different superscripts differ significantly (*p* < 0.05).

## Data Availability

The data presented in this study are available on request from the corresponding author.
